# Three Kampo medicines—bofutsushosan, boiogito, and daisaikoto—have different effects on host fat accumulation and the intestinal microbiota in a high-fat-diet–induced mouse model of obesity

**DOI:** 10.1007/s11418-025-01917-3

**Published:** 2025-06-25

**Authors:** Kosuke Nakamichi, Tetsuhiro Yoshino, Masahiro Akiyama, Aya Jibiki, Yuta Yokoyama, Hitoshi Kawazoe, Sayo Suzuki, Kenji Watanabe, Yun-Gi Kim, Tomonori Nakamura

**Affiliations:** 1 Division of Pharmaceutical Care Sciences, Keio University Graduate School of Pharmaceutical Sciences, 1-5-30 Shibakoen, Minato-ku, Tokyo, 105-8512 Japan; 2 Center for Kampo Medicine, Keio University School of Medicine, 35 Shinanomachi, Shinjuku-ku, Tokyo, 160-8582 Japan; 3 Research Center for Drug Discovery, Keio University Graduate School of Pharmaceutical Sciences, 1-5-30 Shibakoen, Minato-ku, Tokyo, 105-8512 Japan; 4 Division of Pharmaceutical Care Sciences, Center for Social Pharmacy and Pharmaceutical Care Sciences, Faculty of Pharmacy, Keio University, 1-5-30 Shibakoen, Minato-ku, Tokyo, 105-8512 Japan; 5 Department of Microbiology, School of Pharmacy, Kitasato University, 5-9-1 Shirokane, Minato-ku, Tokyo, 108-8641 Japan; 6 Department of Clinical Pharmacy, Keio University School of Medicine, 35 Shinanomachi, Shinjuku-ku, Tokyo, 160-8582 Japan

**Keywords:** Kampo medicine, Bofutsushosan, Boiogito, Daisaikoto, Simultaneous evaluation, Gut microbiota change

## Abstract

Inhibiting body fat accumulation is important for the prevention of obesity. In Japan, three Kampo medicines are commonly used to treat obesity: bofutsushosan, boiogito, and daisaikoto. To compare the influences of these Kampo medicines on the intestinal microbiota, it is necessary to conduct a simultaneous investigation using the same mouse model under the same experimental conditions. C57BL/6J mice were divided into five groups: normal chow (NC), high-fat diet (HFD), HFD + 3% bofutsushosan extract (BTS), HFD + 3% boiogito extract (BOT), and HFD + 3% daisaikoto extract (DST). Epididymal white adipose tissue (WAT) weight, mesenteric WAT weight, serum triglyceride levels, and serum total cholesterol levels were measured. Additionally, total bacteria, alpha diversity, beta diversity, and bacterial composition in stool samples were measured. Body weight and epididymal WAT weight gain were significantly inhibited in the BTS-treated group and DST-treated group, but not in the BOT-treated group, compared with the HFD control group. Additionally, serum total cholesterol levels were significantly lower in the DST-treated group than in the HFD group. Specific intestinal bacteria, *Clostridium sensu stricto 1*, *Erysipelatoclostridium*, *Roseburia*, and the Lachnospiraceae NK4A136 group, were significantly changed in the Kampo-treated groups compared with the HFD group, and each of them was correlated with body weight gain, body fat rate, epididymal WAT weight, or mesenteric WAT weight. Our simultaneous investigation of BTS, BOT, and DST under the same conditions clearly demonstrated different changes in the intestinal microbiota and different effects on fat accumulation as well as their association among the three Kampo medicines.

**Graphical abstract:**

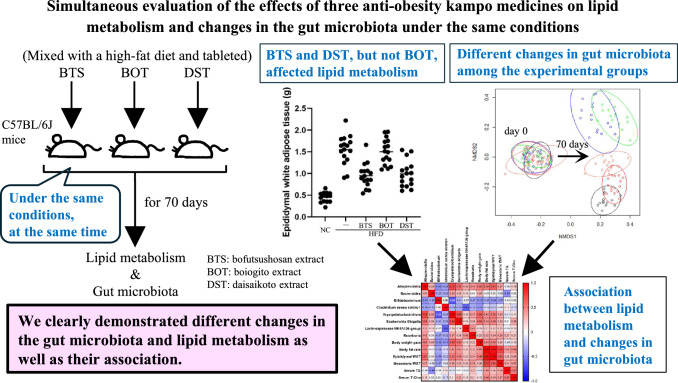

**Supplementary Information:**

The online version contains supplementary material available at 10.1007/s11418-025-01917-3.

## Introduction

The accumulation of body fat is one of the most important factors in the development of obesity. Visceral adipose tissue is a well-known significant risk factor for insulin resistance and the pathogenesis of obesity and obesity-related diseases [1–3]. Therefore, controlling white adipose tissue (WAT) levels is crucial for inhibiting the progression of obesity.

Kampo is a system of traditional medicine that originated in ancient China, was introduced to Japan, and continued to develop independently. Kampo medicines are generally made by combining “crude drugs” derived from plants, animals, or minerals and then extracting the ingredients. In recent years, Kampo medicines have attracted increasing attention [4], which are widely used in Japanese clinics, hospitals, and community pharmacies.

In Japan, three Kampo medicines are commonly used to treat obesity: bofutsushosan, boiogito, and daisaikoto. Previous studies have demonstrated their inhibitory effects on several obesity-related factors, including body weight, WAT weight, and blood glucose levels, in mice [5–7]. A meta-analysis demonstrated that bofutsushosan improves body mass index in patients with obesity [8].

Because of the different composition of their constituent crude drugs, different Kampo medicines are presumed to exert different effects in the clinical settings, even when used to treat the same disease diagnosed according to Western medical theories. In the theory underlying traditional Kampo medicine, this phenomenon has been explained by the relationship between the Kampo medicine and the patient’s constitution, which is called “*Sho*”. For example, bofutsushosan and daisaikoto are thought to be more effective for patients exhibiting the “excess and heat pattern,” whereas boiogito is thought to be more effective for patients exhibiting the “deficiency and cold pattern.” Physicians specializing in Kampo can recognize the effectiveness of different Kampo medicines, even if it may be difficult for medical professionals who are not familiar with Kampo to understand the theory. Uneda et al*.* reported that some physicians who prescribe Kampo medicines recognize the difficulty in following the theory of Kampo due to the lack of scientific evidence [4]. Therefore, it is necessary to investigate why Kampo medicines prescribed for the same indication can have different effects in different patients.

This study focused on the effects of Kampo medicines on the intestinal microbiota, which not only acts on the digestive tract but also exerts a significant influence on the fundamental functions of the body, including nutrition, metabolism, and the immune system [9, 10]. Some previous reports have indicated that there is a relationship between the intestinal microbiota and individual differences in the progression of diseases and the efficacy of various medications in various parts of the body [9, 11, 12]. In the clinical setting, Kampo medicines have been found to have medicinal effects on various areas in the body. Therefore, it is presumed that Kampo medicines affect the intestinal microbiota, which can lead to various therapeutic effects.

Indeed, a relationship between the anti-obesity effects of Kampo medicines and the intestinal microbiota has been reported. Bofutsushosan extract (BTS) exhibits anti-obesity effects as the abundance of *Akkermansia* spp. increases [13, 14]. Moreover, Hussain et al. found that daisaikoto extract (DST) increases the prevalence of several species of intestinal bacteria, including *Roseburia* spp., which were closely related to the anti-obesity effect of DST [15]. Unfortunately, these studies were conducted separately and the relationship between their findings remains unclear. Given that different experimental environments and conditions generally lead to the different compositions of intestinal microbiota, such reports cannot be rigorously compared. Therefore, to clarify this issue, it is necessary to compare the simultaneous changes in the intestinal microbiota among bofutsushosan, boiogito, and daisaikoto, using the same mouse model under the same experimental conditions.

This study focused on the prevention of fat accumulation by BTS, boiogito extract (BOT), and DST by investigating the relationship between their effects on body weight, two types of visceral WAT, lipid metabolism, and changes in the intestinal microbiota in mice.

## Methods

### Materials

BTS, BOT, and DST powders were obtained from TSUMURA & CO. (Tokyo, Japan), with the lot numbers 2220062030, 2220020020, and 2230008010, respectively. The composition of the crude drugs and their amounts are listed in Tables S1–3. NC (AIN-93M) and the HFD (HFD-60) were obtained from Oriental Yeast Co., Ltd. (Tokyo, Japan).

### Three-dimensional high-performance liquid chromatography analysis of Kampo medicine extracts

The three-dimensional high-performance liquid chromatography (HPLC) patterns of each Kampo medicine extract (provided by TSUMURA & CO.) are shown in Fig. S1. For the data acquisition, BTS, BOT, and DST (1.0 g each) were dissolved in 20 mL of methanol, then sonicated for 30 min. The BTS, BOT, and DST samples were obtained by centrifugation at 3000 rpm for 5 min, followed by filtering using a 0.45-µm membrane filter. Then, 30 µL of the sample solution was injected into the HPLC machine (LC 10A; injector, CTO-10AC; analysis software, CLASS-M10A ver. 1.64; Shimadzu Corp., Kyoto, Japan). A TSK-GEL 80TS column (250 × 4.6 mm i.d.; Tosho Co., Tokyo, Japan) was used for analysis. The mobile phase consisted of (A) 0.05 M ammonium acetate (pH 3.6) and (B) 100% acetonitrile. The gradient dilution was set as follows: A:B = 90:10 (0 min)–0:100 (60 min), linear gradient, then 100% B for 20 min. The flow rate was 1.0 mL/min, and the column temperature was 40 °C. Peaks were detected using a photodiode array detector (UV length 200–400 nm; SPD-M10AVP; Shimadzu Corp.).

### Mouse experiment

All experimental procedures were approved by the Laboratory Committee of Keio University (Approval No. A2022-181). Eighty 4-week-old male C57BL/6 J mice were purchased from Clea Japan (Shizuoka, Japan). After acclimatization for 1 week, the mice were divided into five groups: (1) NC; (2) HFD; (3) HFD + 3.0% BTS (HFD + BTS group); (4) HFD + 3.0% BOT (HFD + BOT group); and (5) HFD + 3.0% DST (HFD + DST group). To avoid negative influences on lipid metabolism due to social isolation [16], each cage accommodated four mice. In our experiments, the mice were administered Kampo medicine together with a HFD. This is thought to cause the mice less stress compared with oral gavage. The administered dosage was largely consistent with that used in previous studies [5, 13]. The composition of each diet is listed in Table 1. All mice were housed for 10 weeks. The experimental room was kept at a temperature of 22–24 °C on a 12-h light/dark cycle. The mice were allowed free access to water and food.

**Table 1:** Composition of the experimental diets

	NC (%)	HFD (%)	HFD + BTS (%)	HFD + BOT (%)	HFD + DST (%)
Milk casein	14	25.6	24.8	24.8	24.8
L-Cysteine	0.18	0.36	0.349	0.349	0.349
Maltodextrin	0	6	5.82	5.82	5.82
Corn starch	46.6	0	0	0	0
Alpha corn starch	15.5	16	15.5	15.5	15.5
Sucrose	10	5.5	5.34	5.34	5.34
Soybean oil	4	2	1.94	1.94	1.94
Lard	0	33	32.0	32.0	32.0
Cellulose	5	6.61	6.41	6.41	6.41
Calcium carbonate	0	0.18	0.175	0.175	0.175
Choline bitartrate	0.25	0.25	0.243	0.243	0.243
Tertiary butylhydroquinone	< 0.01	0	0	0	0
AIN-93G vitamin mix	1	1	0.97	0.97	0.97
AIN-93G mineral mix	3.5	3.5	3.40	3.40	3.40
BTS	0	0	3	0	0
BOT	0	0	0	3	0
DST	0	0	0	0	3
Total	100	100	100	100	100

Body weight was measured once a week, and all feed was changed twice a week. The weight of the food in the cage was measured at the time of food change, and food intake was calculated as the amount of reduction in the weight of the food per cage. For each experimental group, the average food intake (per day, per mouse) was calculated by dividing the food intake by the number of mice and interval days in the cages. On days 0 and 70, fecal samples were collected directly from the anus of each mouse without prior fasting. At the end of the experiment, each mouse was euthanized through cervical dislocation under anesthesia. The blood of each mouse was collected, and the weight of epididymal and mesenteric WAT was measured. Feces, serum, and WAT were all stored at − 80 °C until the next measurement was performed. Serum triglyceride and total cholesterol levels were measured using LabAssay™ Triglyceride and LabAssay™ Cholesterol, respectively (Fujifilm Wako Pure Chemical, Osaka, Japan).

An oral glucose tolerance test (OGTT) was conducted in the 9th week of the experimental period. The mice were fasted overnight before the OGTT, and were administered 2.0 g/kg glucose via oral gavage. Glucose levels at 0, 30, 60, 120, and 180 min after glucose administration were measured using STAT STRIP Xpress 900 (Nova Biomedical, Waltham, MA).

### Gut microbiota analysis

DNA from the intestinal microbiota was extracted from 200 mg mouse stool samples using the following procedures. Stool samples were smashed with glass beads (BioSpec Products, Bartlesville, OK) and 540 µL SLX-Mlus Buffer (Omega Bio-Tek, Inc., Norcross, GA). Then, 60 µL DS buffer (Omega Bio-Tek, Inc.) and 20 µL proteinase K (Omega Bio-Tek, Inc.) were added to each sample. After vortexing, each sample was incubated at 70 °C for 10 min and then at 95 °C for 5 min. Subsequently, 200 µL SP2 buffer (Omega Bio-Tek, Inc.) was added to each sample and vortexed vigorously for 30 s. After cooling on ice for at least 5 min, each sample was centrifuged at 13,000×*g*, 4 °C, for 7 min. Then, 200 µL supernatant was isolated as a DNA solution. Extracted DNA was purified using MagLEAD (Precision System Science, Pleasanton, CA). Purified DNA was used for sequencing with MiSeq. Alpha diversity (Pielou’s evenness index), beta diversity (Jaccard distance), and the ratio of each bacterium was calculated using QIIME2.

Total bacteria were measured using the following procedure. Stool samples were smashed with glass beads (BioSpec Products), 50 µL of 10% SDS (Nippon Gene, Tokyo, Japan), 500 µL TE-saturated phenol (Nacalai Tesque, Kyoto, Japan), and extraction buffer (prepared following a previous report [17]). After centrifuging at 20,000×*g* for 5 min, 400 µL supernatant and 400 µL phenol:chloroform:isoamyl alcohol 25:24:1 solution (Nacalai Tesque) were mixed. After centrifuging at 20,000×*g* for 5 min, 250 µL supernatant and 25 µL of 3 M sodium acetate (Nippon Gene) were mixed. Then, each sample was washed with 250 µL isopropanol (Nacalai Tesque) followed by 500 µL of 70% ethanol. Each extracted DNA sample was resolved in 1 mL TE buffer (SERVA Electrophoresis GmbH, Heidelberg, Germany). Next, 2 µL of the sample solution was mixed with a reaction buffer composed of 10 µL TB Green (Takara Bio, Shiga, Japan), 0.2 µL forward and reverse primers, respectively, and 7.6 µL DNase-free H_2_O (Invitrogen, Carlsbad, CA). The primers and amplification algorithm followed a previous report [17].

### Statistical analysis

Data are shown as means ± standard deviation. Adipose tissue weight and total bacteria were compared by one-way analysis of variance (ANOVA) followed by Tukey’s test. Body weight was analyzed by two-way ANOVA. The ratio of intestinal bacteria was first checked by a histogram and the Shapiro–Wilk test to determine if it followed a Gaussian distribution. After that, it was compared by one-way ANOVA followed by Tukey’s test and Pearson correlation coefficient in the case of a Gaussian distribution, while the Kruskal–Wallis and the Dunn tests (Bonferroni correction) and Spearman correlation coefficient were used for non-Gaussian distributions. All analyses were performed by Prism 9 (GraphPad Software Inc., San Diego, CA), and data with a *p* value lower than 0.05 were considered significant.

## Results

**Fig. 1:**
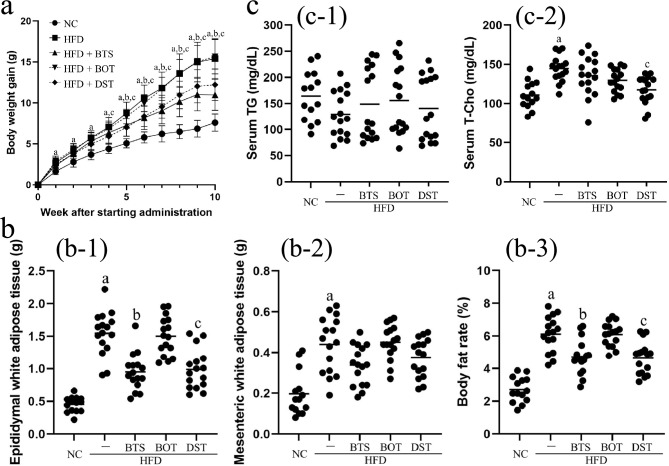
BTS and DST affect body weight and lipid metabolism. **a** Body weight gain after starting administration of the Kampo medicines. Statistical analysis was performed by two-way analysis of variance (ANOVA). **b** Epididymal white adipose tissue (WAT) weight (b-1), mesenteric WAT weight (b-2), and body fat rate (b-3) at day 70 were assessed by ANOVA. Body fat rate was calculated as: ([epididymal WAT weight] + [mesenteric WAT weight]) / (body weight) × 100, at day 70. **c** Serum triglyceride (TG, c-1) and serum total cholesterol (T-Cho, c-2) levels at day 70 were assessed by ANOVA. *n* = 14–16 per group. **a** *p* < 0.05 (NC vs. HFD); **b** *p* < 0.05 (HFD vs. HFD + BTS); **c** *p* < 0.05 (HFD vs. HFD + DST). *BTS* bofutsushosan extract, *BOT* boiogito extract, *DST* daisaikoto extract, *HFD* high-fat diet, *NC* normal chow. Each Kampo extract was mixed into HFD at a ratio of 3%

During the experiments, mice in the HFD group gained significantly more body weight compared with those in the NC group. The HFD + BTS and HFD + DST groups showed significantly less weight gain compared with the HFD group after 5 and 4 weeks, respectively (Fig. 1a). Average food intakes per mouse in the NC, HFD, HFD + BTS, HFD + BOT, and HFD + DST groups were 2.5, 2.3, 2.1, 2.3, and 2.2 g/day, respectively. The HFD group showed a significant increase in the body fat rate, epididymal WAT weight, and mesenteric WAT weight compared with the NC group. The HFD + BTS and HFD + DST groups showed a significantly lower body fat rate and epididymal WAT weight compared with the HFD group. There was a slight decrease in mesenteric WAT weight in the HFD + BTS and HFD + DST groups (Fig. 1b). Serum triglyceride levels did not change significantly in the HFD + BTS, HFD + BOT, and HFD + DST groups. In contrast, serum total cholesterol levels increased by 1.5-fold in the HFD group compared with the NC group (*p* < 0.01), and the HFD + DST group showed significantly decreased serum total cholesterol levels compared with the HFD group (Fig. 1c). In addition, there was no significant change in the OGTT scores among the five groups (data not shown). These results suggest that BTS and DST had significant inhibitory effects on fat accumulation caused by feeding the mice a HFD.

**Fig. 2:**
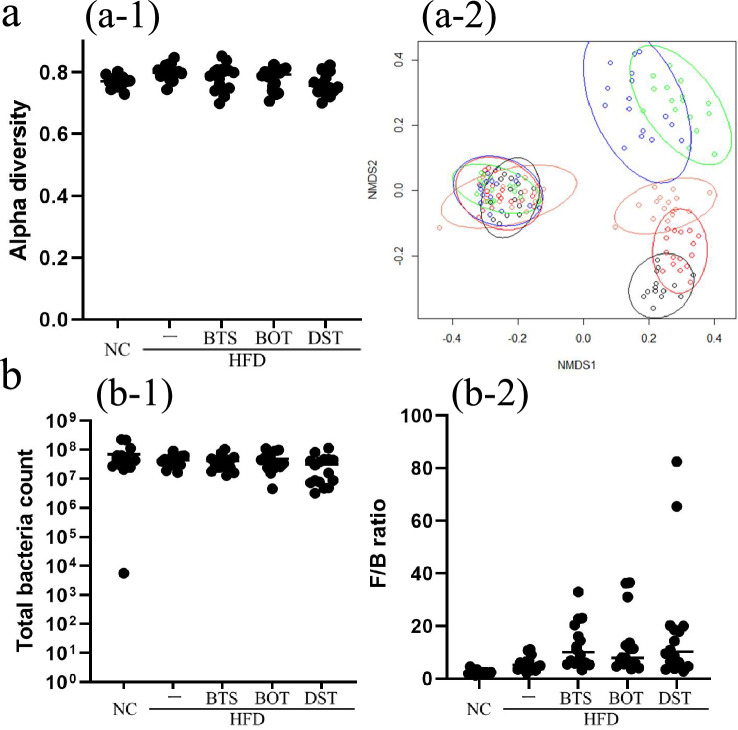
BTS, BOT, and DST affect the intestinal microbiota at day 70. **a** Pielou’s evenness index (alpha diversity, a-1, assessed by ANOVA) and Jaccard distance (beta diversity, a-2) at days 0 and 70. In the figure of Jaccard distance, the left overlapping clusters represent day 0 and the clusters on the right side represent day 70. Black: NC group; red: HFD group; green: HFD + BTS group; orange: HFD + BOT group; blue: HFD + DST group. **b** Total bacterial count was assessed by ANOVA (b-1), and the Firmicutes/Bacteroidetes (F/B) ratio was assessed by the Kruskal–Wallis test (b-2). *n* = 14–16 per group. BTS, bofutsushosan extract; BOT, boiogito extract; DST, daisaikoto extract; HFD, high-fat diet; NC, normal chow. Each Kampo extract was mixed into HFD at a ratio of 3%

BTS, BOT, and DST appeared to have different effects on the intestinal microbiota of mice. Despite there being no significant changes in alpha diversity between each group, we found different clusters not only between day 0 and day 70 but also among each group at day 70 for beta diversity (Fig. 2a). These data indicate that the composition of the intestinal microbiota changed during the experimental period, and specific intestinal microbiota were established depending on the diet and Kampo medicines at the end of the experiment although there were no significant differences in the total number of bacteria and the Firmicutes/Bacteroidetes ratio (Fig. 2b).

**Fig. 3:**
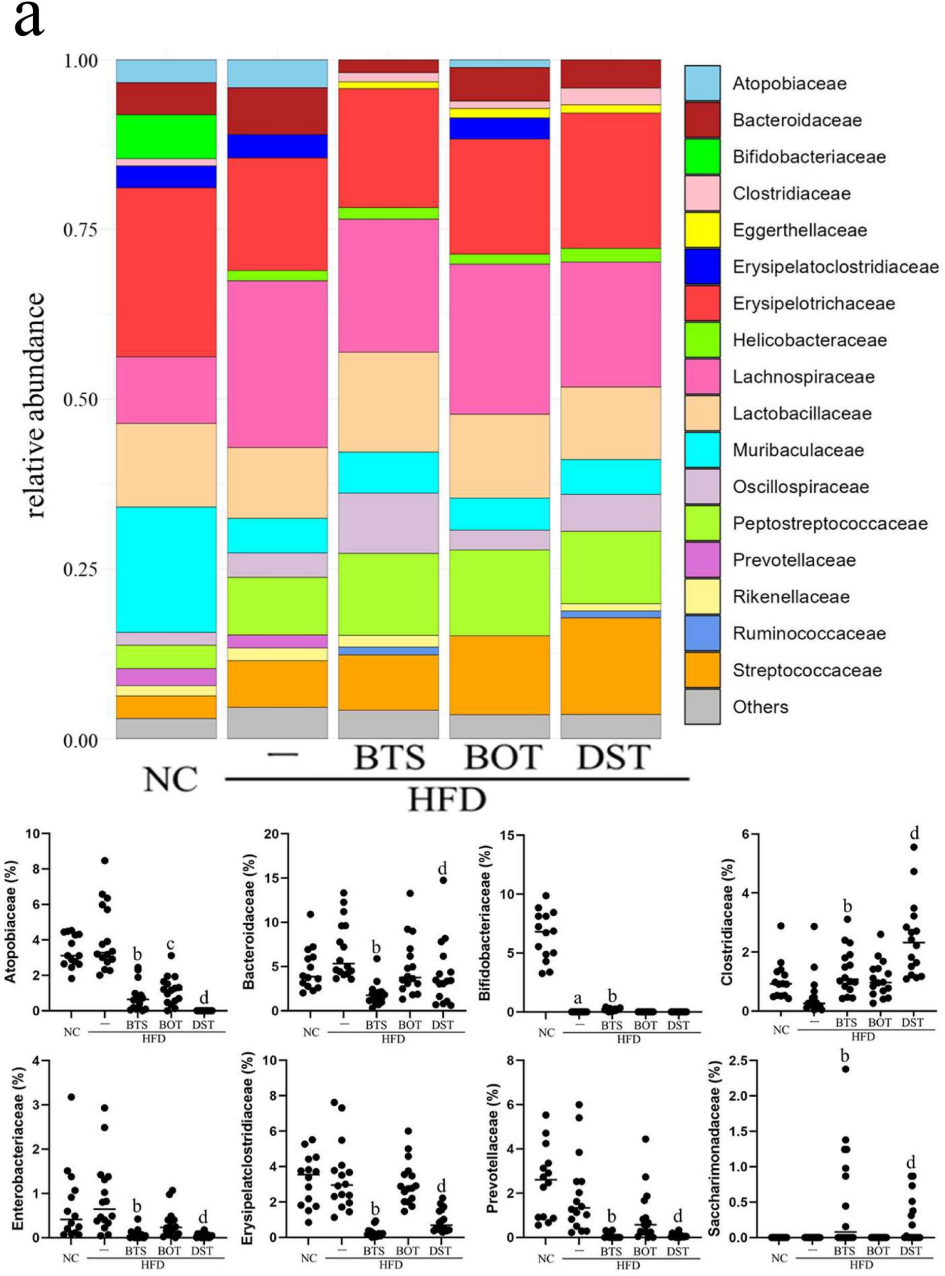

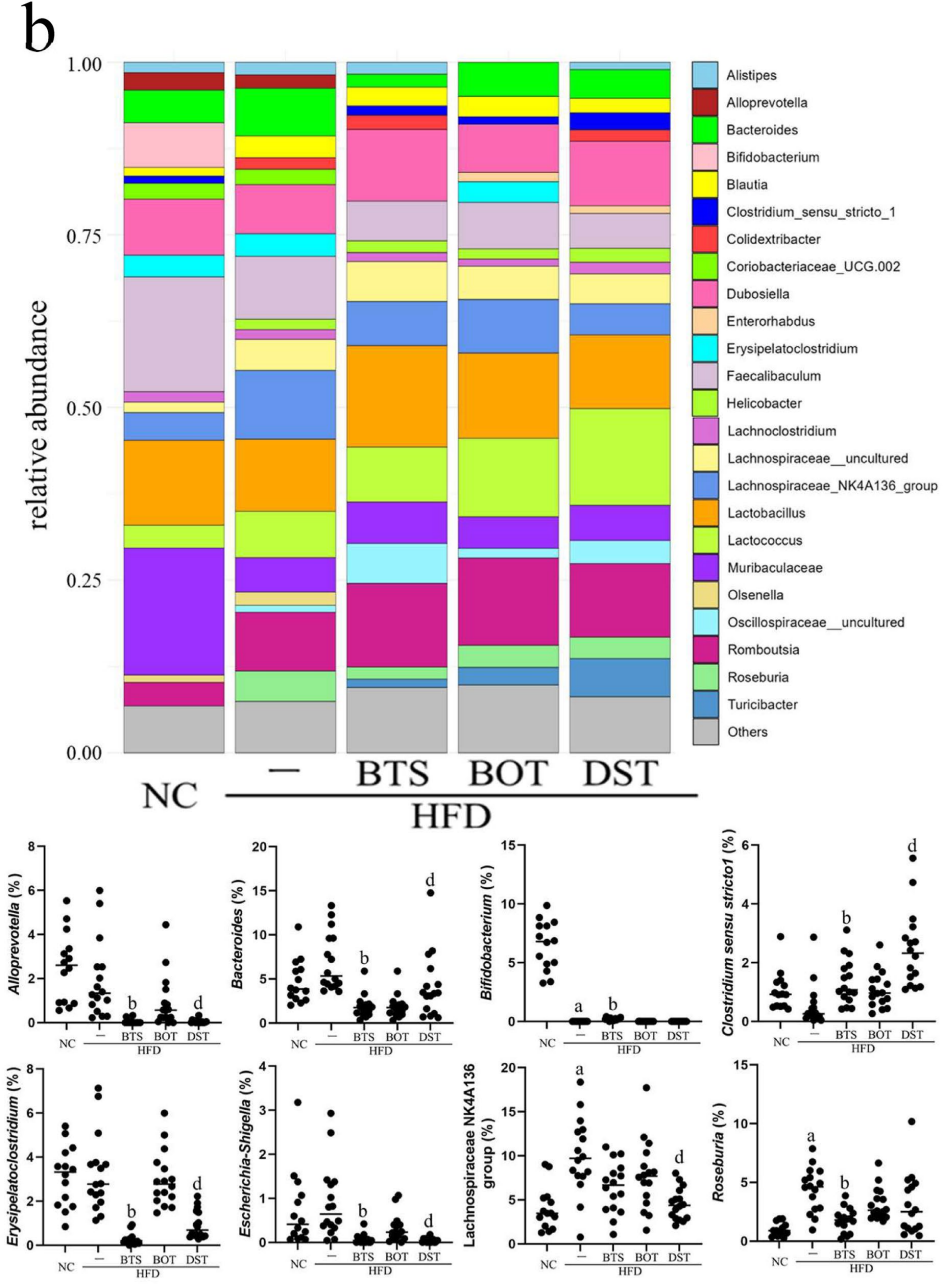
Composition of the intestinal microbiota at day 70. **a** Major bacterial composition at the phylum level on day 70. **b** Major bacterial composition at the genus level at day 70. Statistical analysis of the bacterial ratio was performed by the Kruskal–Wallis test. *n* = 14–16 per group. **a** *p* < 0.05 (NC vs. HFD); **b** *p* < 0.05 (HFD vs. HFD + BTS); **c** *p* < 0.05 (HFD vs. HFD + BOT); d: *p* < 0.05 (HFD vs. HFD + DST). *BTS* bofutsushosan extract, *BOT* boiogito extract, *DST* daisaikoto extract, *HFD* high-fat diet, *NC* normal chow. Each Kampo extract was mixed into HFD at a ratio of 3%

At the phylum level (Fig. 3a), a significantly lower ratio of Atopobiaceae was found in the HFD + BTS, HFD + BOT, and HFD + DST groups than in the HFD group. Clostridiaceae was significantly increased in the HFD + BTS and HFD + DST groups compared with the HFD group. In addition, Bacteroidaceae, Enterobacteriaceae, Erysipelatoclostridiaceae, Prevotellaceae, and Saccharimonadaceae were significantly reduced in the HFD + BTS and HFD + DST groups compared with the HFD group. At the genus level (Fig. 3b), *Clostridium sensu stricto 1* was significantly increased and *Alloprevotella*, *Bacteroides*, *Erysipelatoclostridium*, and *Escherichia-Shigella* were significantly reduced in the HFD + BTS and HFD + DST groups compared with the HFD group.

Moreover, we found specific microbiota for which the ratio was significantly improved by only one Kampo medicine (Fig. 3). Bifidobacteriaceae and *Bifidobacterium* were significantly reduced in the HFD group compared with the NC group. However, the Bifidobacteriaceae ratio was restored only in the HFD + BTS group compared with the HFD group (*p* < 0.001). The ratios of *Roseburia* and the Lachnospiraceae NK4A136 group were significantly higher in the HFD group compared with the NC group. The ratio of *Roseburia* was significantly reduced only in the HFD + BTS group compared with the HFD group. In contrast, the ratio of the Lachnospiraceae NK4A136 group was significantly lower only in the HFD + DST group compared with the HFD group.

**Fig. 4:**
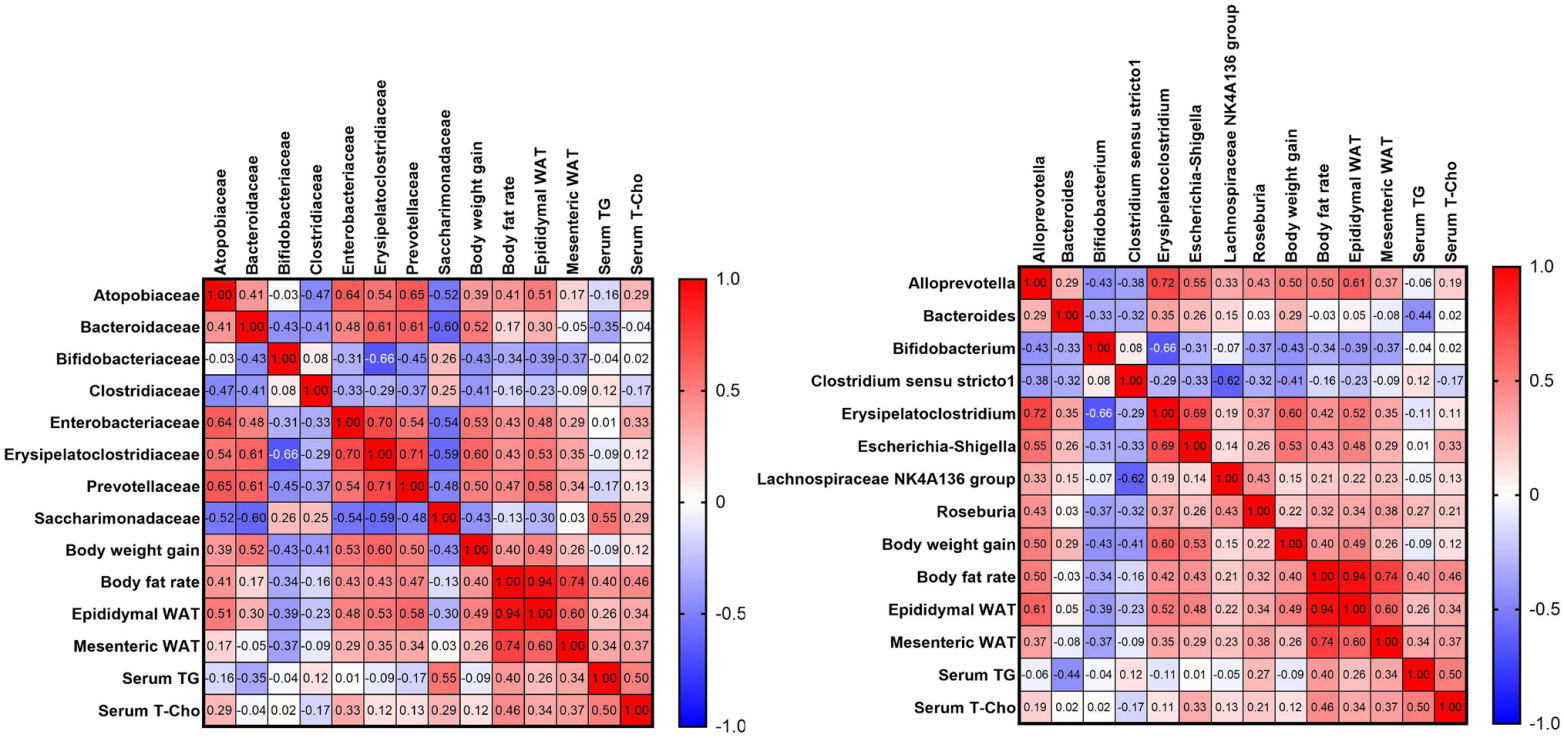
Heat map of Spearman correlation analysis. The numbers show the correlation coefficients. *n* = 14–16 per group. **a** *p* < 0.05 (NC vs. HFD); **b** *p* < 0.05 (HFD vs. HFD + BTS); **c** *p* < 0.05 (HFD vs. HFD + BOT); **d** *p* < 0.05 (HFD vs. HFD + DST). *BTS* bofutsushosan extract, *BOT* boiogito extract, *DST* daisaikoto extract, *HFD* high-fat diet, *NC* normal chow. Each Kampo extract was mixed into HFD at a ratio of 3%

Spearman correlation analysis among the HFD-fed mice (Fig. 4) showed that the ratios of Atopobiaceae, Enterobacteriaceae, Erysipelatoclostridiaceae, Prevotellaceae, *Alloprevotella*, *Erysipelatoclostridium*, and *Escherichia-Shigella* were positively correlated with body weight gain, body fat rate, and epididymal WAT weight. The ratios of Bifidobacteriaceae and *Bifidobacterium* were negatively correlated with body weight gain, body fat rate, epididymal WAT weight, and mesenteric WAT weight. In addition, the ratios of Clostridiaceae and *Clostridium sensu stricto 1* were negatively correlated with body weight gain. Of note, the ratios of *Roseburia* and the Lachnospiraceae NK4A136 group had weak positive correlations with body weight gain, body fat rate, epididymal WAT weight, and mesenteric WAT weight.

## Discussion

Our data indicate that BTS and DST inhibit fat accumulation in correlation with changes in the intestinal microbiota. In addition, only the HFD + DST group showed significantly lower serum total cholesterol levels compared with the HFD group. Although there are no reports showing that DST inhibits HMG-CoA reductase activity, a previous study reported that DST had an inhibitory effect on pancreas lipase activity [18], which suggests that the mechanism underlying the lower serum cholesterol levels observed in the HFD + DST group in the present study might be the inhibition of cholesterol absorption. However, it should be noted that there were no significant differences in the OGTT score among the experimental groups. We were unable to elucidate the reason for this lack of an effect on glucose metabolism, but it is possible that the mice were too young for their glucose metabolism to be evaluated. Our experiments involved 4-week-old mice. Considering that a previous study reported that 3-week-old mice exhibit better glucose tolerance compared with 12-week-old mice despite HFD feeding [19], it might have been difficult for such young mice to develop HFD-induced insulin resistance. Therefore, a future experiment using older mice should be considered.

In contrast, the BOT did not show a significant effect on body weight or WAT weight. Our study indicates that although the BOT was ineffective, the BTS and DST were effective at reducing fat accumulation in the current mouse model, even though all three Kampo medicines are used to treat obesity in humans. *Scutellaria baicalensis* and *Rheum palmatum*, which are commonly included in BTS and DST but not in BOT, have been previously studied for their effect on body weight and adipose tissue weight in mice. Extract of *S. baicalensis* is reported to reduce body weight and adipose tissue weight in C57BL/6J mice with diet-induced obesity [20]. Baicalin, one of the major natural components in *S. baicalensis* is reported to reduce body weight and WAT weight through the induction of thermogenesis in adipose tissue [21]. Previous studies have reported that rhein and emodin, two of the main components of *R. palmatum*, have an abatement effect on the body weight and the adipose tissue weight in obese C57BL/6J mice [22, 23]. These crude drugs are assumed to have contributed to the disparity in reduction effects on body weight and WAT weight between BTS/DST and BOT observed in the present study. In future, more detailed identification of the crude drugs that contribute to the effect of each Kampo medicine is needed. Another possibility is that an alternative mouse model related to obesity might yield different results about the effect of these Kampo medicines. BOT may have been ineffective because the mouse model we used differed from that in a previous report using a spontaneous obese-type II diabetes mellitus mouse model to show that BOT exerts an anti-obesity effect [6]. Using a variety of mouse models would enable us to more rigorously analyze the differences in the effects of BTS, BOT, and DST on body weight and WAT weight.

This study observed changes in specific intestinal microbiota. Beta diversity (Jaccard distance) suggested that a different composition of the intestinal microbiota was created in each experimental group on day 70. However, alpha diversity (Pielou’s evenness index) and total bacteria count indicated that the HFD and Kampo medicines did not affect the intestinal microbiota drastically. Of all the intestinal microbes detected in this experiment, the HFD + BTS and HFD + DST groups showed significant increases of Clostridiaceae and *Clostridium sensu stricto 1*, and significant decreases of Bacteroidaceae, *Bacteroides*, Erysipelatoclostridiaceae, *Erysipelatoclostridium*, Enterobacteriaceae, *Escherichia-Shigella*, Prevotellaceae, *Alloprevotella*, and Saccharimonadaceae compared with the HFD group. These microbes may partially explain the distinctive effects of the BTS and DST compared with the BOT. *Clostridium sensu stricto 1* is reported to be a butyrate-producing bacterium [24]. Li et al*.* demonstrated that genistein, an isoflavone in the flowering plant *Gardenia jasminoides*, significantly restores *Clostridium sensu stricto 1* levels in C57BL/6J mice fed an HFD [25]. Sang-Hyun et al*.* proved that *Bacteroides thetaiotaomicron* is an inducer of HFD-induced obesity, affecting lipid metabolism [26]. Guo et al*.* found that the administration of ougan (*Citrus reticulata* cv. Suavissima) juice reduces Erysipelatoclostridiaceae and *Erysipelatoclostridium* levels in correlation with total cholesterol and epididymal WAT weight [27]. Yang et al*.* pointed out that *Escherichia-Shigella* is a pro-inflammatory bacterium, and dietary methionine significantly increases its abundance, which may be one of the risk factors for cognitive dysfunction [28]. Therefore, these microbes may have a key role in the effects observed in the HFD + BTS and HFD + DST groups. However, several studies have reported that *Alloprevotella* might have a suppressive effect on fat accumulation and obesity [29, 30]. Nevertheless, further research is still needed.

Taken together, 11 groups of bacteria including *Clostridium sensu stricto 1* significantly changed in HFD + BTS and HFD + DST groups, while HFD + BOT group had no significant changes in these bacteria. In Kampo medicine, bofutsushosan and daisaikoto are thought to have medicinal effects for patients exhibiting the “excess and heat pattern,” while boiogito is thought to be effective for patients exhibiting the “deficiency and cold pattern” in the clinical setting. It is notable that these Kampo medicines affected the intestinal microbiota in clearly different ways, considering how they traditionally distinguished in the theory of Kampo medicine. Although this one study cannot be used to draw a definitive conclusion, the results suggest that these bacteria account for the “excess and heat pattern” and the “deficiency and cold pattern” of the above Kampo medicines. Indeed, in a prospective observational study for patients at several Kampo clinics, Maeda-Minami points out that BMI and blood pressure are among the most important factors in discriminating between the excess and deficiency patterns [31, 32]. In addition, some reports mention that *Erysipelatoclostridium ramosum* and *Escherichia-Shigella* are associated with BMI [33, 34]. Other reports suggest that *Escherichia-Shigella*, Saccharimonadaceae, and bacteria-produced butyrate are associated with blood pressure [35, 36]. Considering the above, these bacteria may be associated with the effects of BTS and DST on obesity with the “excess pattern.” However, further validation in the clinical setting is needed.

We observed other intestinal microbes that were significantly changed by only one of the three Kampo medicines. In the HFD + BTS group, Bifidobacteriaceae and *Bifidobacterium* were significantly elevated and *Roseburia* was significantly reduced compared with the HFD group. The abundance of the Lachnospiraceae NK4A136 group, which was significantly increased by the HFD, was significantly reduced in the HFD + DST group. These changes imply a significant difference between the effects of the BTS and DST. Wang et al*.* found that resveratrol, a natural polyphenol, reduces the abundance of the Lachnospiraceae NK4A136 group, which is associated with obesity [37]. Contrary to our results, a previous report indicated that *Roseburia* spp. is a producer of short-chain fatty acids [28]. Further research is needed to clarify the details of these changes to the intestinal microbiota and their influence on fat accumulation, including short-chain fatty acids and other metabolites.

Akkermansiaceae and *Akkermansia* were not detected in the HFD-fed mice, including the HFD + BTS group, in contrast to the findings of Fujisaka et al. and Nishiyama et al. [13, 14]. In addition, the abundance of *Roseburia* and *Lactobacillus* was not significantly changed in the HFD + DST group compared with the HFD group, contrary to the results of Hussain et al. [15]. This might be due to variance in experimental mouse models and the environments in which the mice were raised (for example, climate and food). Further studies are needed to resolve the conflicts between changes to the gut microbiota and their underlying mechanisms.

This study has several limitations that should be considered. First, there was no experimental group combining NC with BTS, BOT, or DST. Therefore, it cannot be definitively concluded that the inhibitory effects of BTS, BOT, or DST on fat accumulation are independent of the HFD. Second, contribution and dependence of the intestinal microbiota on the inhibitory effect of BTS and DST on WAT are yet to be determined. It will be necessary to clarify the detailed relationship between the effects of BTS and DST by observing the effects of administering antibiotics or fecal microbiota transplantation. Third, the influence of the Kampo medicines on other organs except for epididymal WAT and mesenteric WAT was not examined. Future studies should clarify their effect on, for example, brown adipose tissue, hepatic tissue, and other organs.

To our knowledge, this is the first report to clarify the anti-obesity effects of multiple Kampo medicines and changes in the intestinal microbiota in one mouse model at the same time. Our results from the simultaneous evaluation of three types of anti-obesity Kampo medicine suggest that among the three major Kampo medicines used to treat obesity in Japan, BTS and DST, but not BOT, may inhibit body weight gain and fat accumulation in the present HFD-induced mouse model of obesity. The observed changes in the intestinal microbiota may be an important factor underlying the effects of BTS, BOT, and DST. The differences in changes in the intestinal microbiota and the effects on lipid metabolism among the three Kampo medicines as well as the association between them were more clearly demonstrated by our experiment using all three drugs simultaneously under the same conditions. Interestingly, it is possible that the differences in changes in the intestinal microbiota observed in this study might be able to account for the differences between excess and deficiency patterns in the effects of Kampo medicines. Further research on the detailed pharmacological mechanisms and further clinical validation are needed.

## Supplementary Information

Below is the link to the electronic supplementary material.

Supplementary file1 (DOCX 1980 kb)
