# Supplementary material for: Three Kampo medicines—bofutsushosan, boiogito, and daisaikoto—have different effects on host fat accumulation and the intestinal microbiota in a high-fat-diet–induced mouse model of obesity
Source: J Nat Med. 2025 Jun 25;79(5):1044–56. doi: 10.1007/s11418-025-01917-3 (PMC13035580; doi:10.1007/s11418-025-01917-3)
Supplement: Supplementary file 1 — Supplementary file1 (DOCX 1980 kb) [file 11418_2025_1917_MOESM1_ESM.docx]

**Supplemental Data**

**Tables**

**Table S1.** The composition of crude drugs in BTS (4.5 g)

| Aluminum Silicate Hydrate with Silicon Dioxide | 3.0 g |
| --- | --- |
| Gypsum | 2.0 g |
| Glycyrrhiza | 2.0 g |
| Scutellaria Root | 2.0 g |
| Platycodon Root | 2.0 g |
| Atractylodes Rhizome | 2.0 g |
| Rhubarb | 1.5 g |
| Ephedra Herb | 1.2 g |
| Saposhnikovia Root and Rhizome | 1.2 g |
| Schizonepeta Spike | 1.2 g |
| Mentha Herb | 1.2 g |
| Forsythia Fruit | 1.2 g |
| Gardenia Fruit | 1.2 g |
| Cnidium Rhizome | 1.2 g |
| Peony Root | 1.2 g |
| Japanese Angelica Root | 1.2 g |
| Sodium Sulfate Hydrate | 0.7 g |
| Ginger | 0.3 g |

**Table S2.** The composition of crude drugs in BOT (3.75 g)

| Astragalus Root | 5.0 g |
| --- | --- |
| Sinomenium Stem and Rhizome | 5.0 g |
| Atractylodes Rhizome | 3.0 g |
| Jujube | 3.0 g |
| Glycyrrhiza | 1.5 g |
| Ginger | 1.0 g |

**Table S3.** The composition of crude drugs in DST (4.5 g)

| Bupleurum Root | 6.0 g |
| --- | --- |
| Pinellia Tuber | 4.0 g |
| Scutellaria Root | 3.0 g |
| Peony Root | 3.0 g |
| Jujube | 3.0 g |
| Immature Orange | 2.0 g |
| Ginger | 1.0 g |
| Rhubarb | 1.0 g |


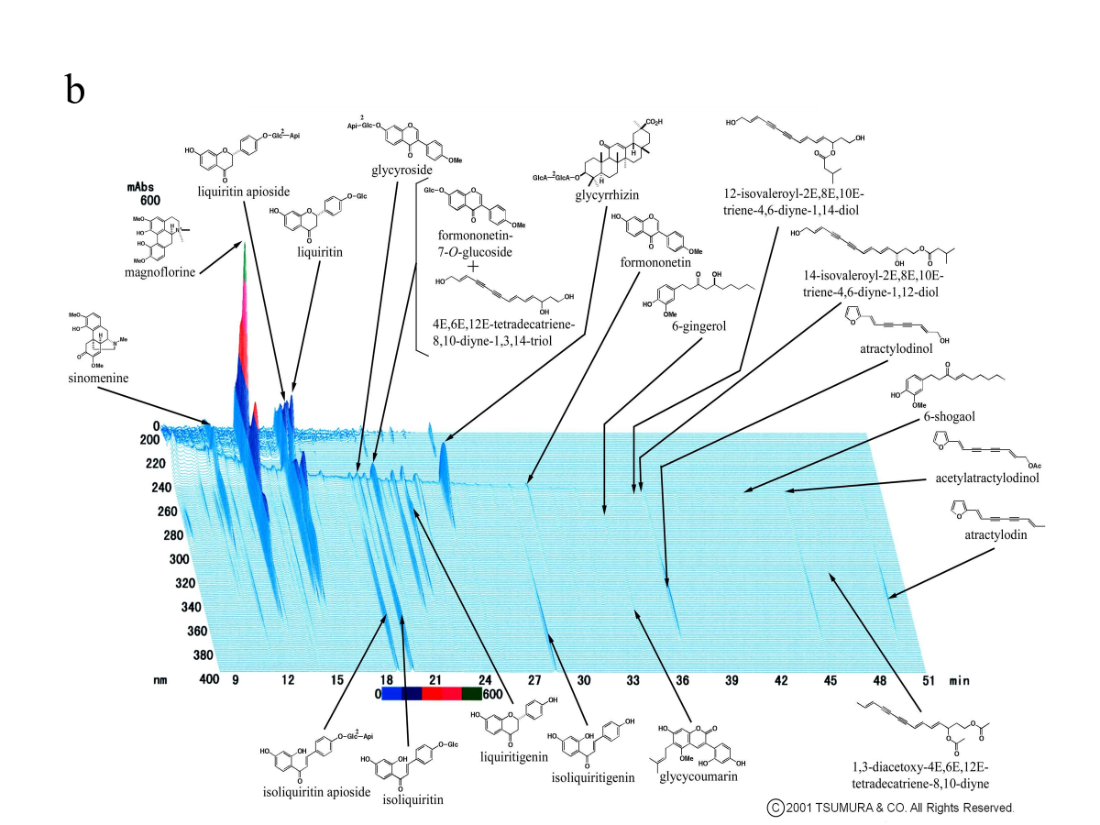

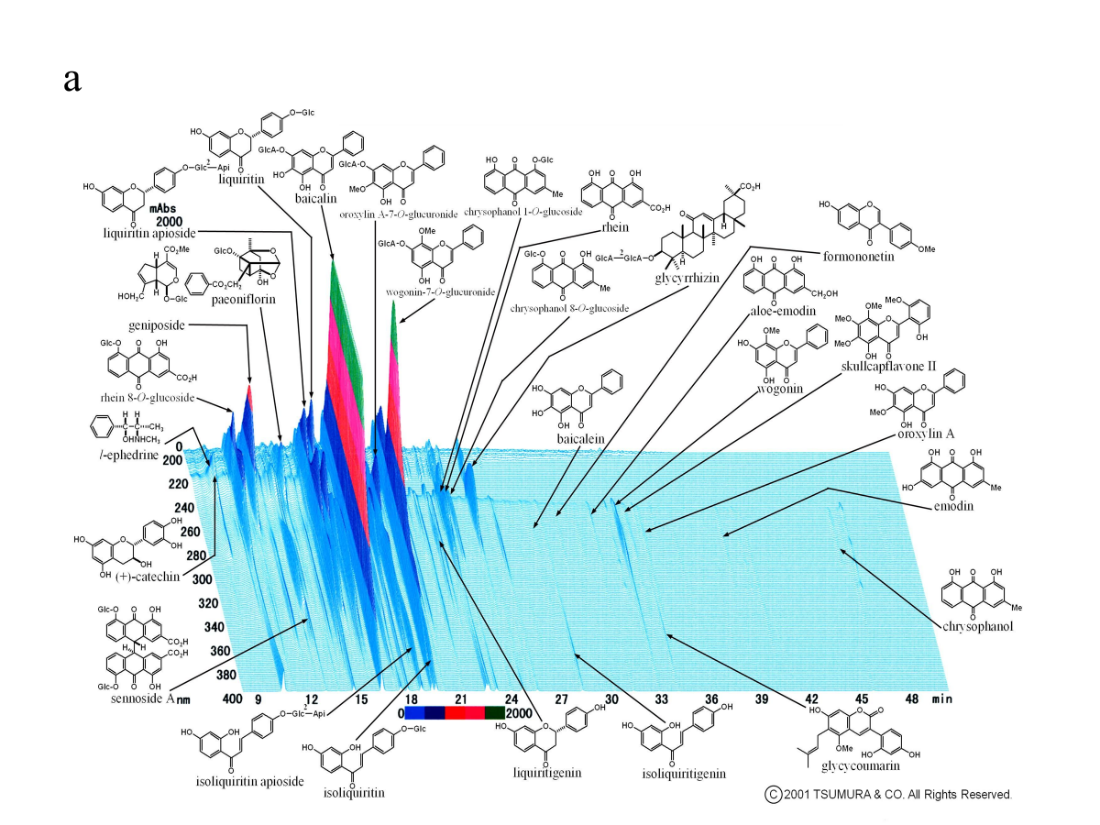
**Figure**


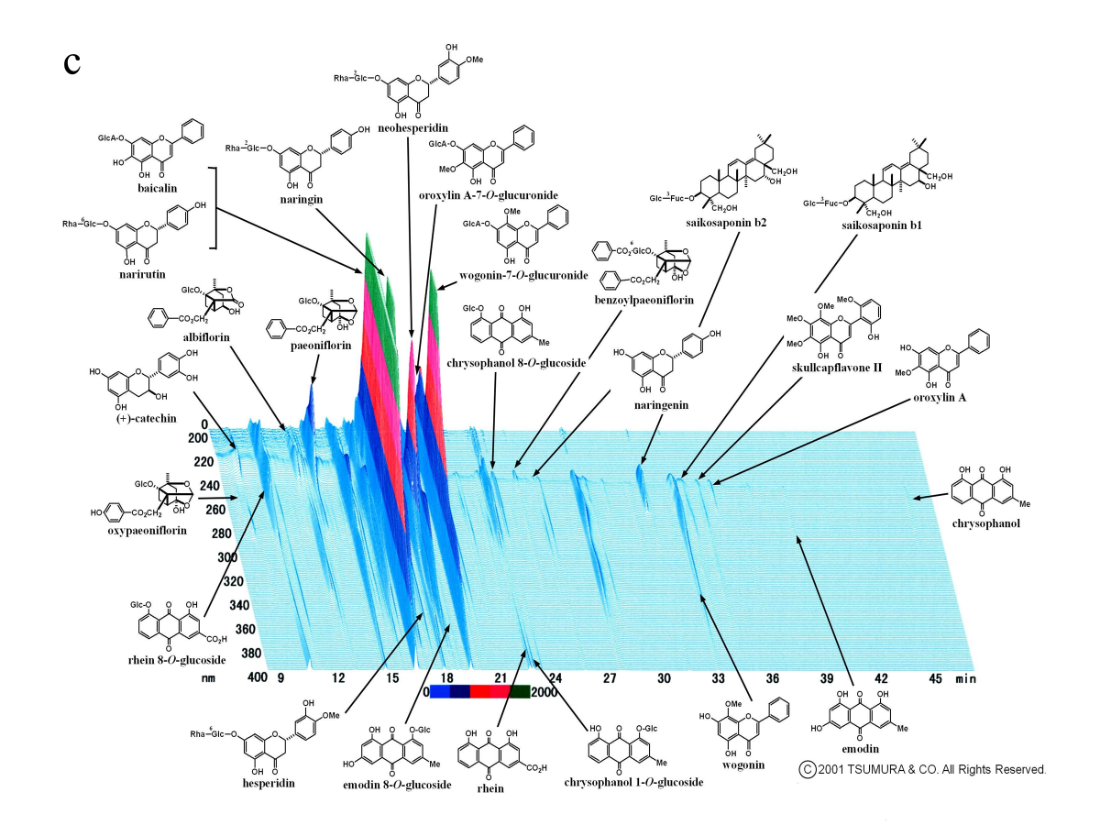


**Fig. S1.** Three-dimensional high-performance liquid chromatography patterns of BTS (Lot No. 2220062030), BOT (Lot No. 2220020020), and DST (Lot No. 2230008010). **a**: BTS. **b**: BOT. **c**: DST.

BTS, bofutsushosan extract; BOT, boiogito extract; DST, daisaikoto extract.
